# Antioxidant Activity and Inhibition of Digestive Enzymes of New Strawberry Tree Fruit/Apple Smoothies

**DOI:** 10.3390/antiox12040805

**Published:** 2023-03-26

**Authors:** Katarzyna Angelika Gil, Paulina Nowicka, Aneta Wojdyło, Gabriele Serreli, Monica Deiana, Carlo Ignazio Giovanni Tuberoso

**Affiliations:** 1 Department of Life and Environmental Sciences, University of Cagliari, Cittadella Universitaria di Monserrato, S.P. Monserrato-Sestu km 0.700, 09042 Monserrato, Italy; 2 Department of Fruit, Vegetable and Plant Nutraceutical Technology, Wrocław University of Environmental and Life Sciences, 37 Chełmońskiego Street, 51-630 Wroclaw, Poland; 3 Department of Biomedical Sciences, University of Cagliari, Cittadella Universitaria, SS 554, km 4.5, 09042 Monserrato, Italy

**Keywords:** antioxidant activity, digestive enzymes, *Arbutus unedo*, Caco-2 cell line, HPLC-ELSD, LC-PDA/MS QTof, phenolic compounds, smoothies, UPLC-PDA

## Abstract

In this study, original smoothies obtained with strawberry tree fruit puree and apple juice enriched with *Diospyros kaki* fruits, *Myrtus communis* purple berry extract, *Acca sellowiana*, and *Crocus sativus* petal juice were evaluated for their antioxidant activity and inhibition of targeted digestive enzymes. Values of CUPRAC, FRAP, ORAC, DPPH^•^, and ABTS^•+^ assays generally increased with plant enrichment, particularly for *A. sellowiana* addition (ABTS^•+^ 2.51 ± 0.01 mmol Trolox/100 g fw). The same trend was observed regarding the ability to scavenge reactive oxygen species (ROS) tested in Caco-2 cell cultures. Inhibitory activity on α-amylase and α-glucosidase was increased by *D. kaki*, *M. communis*, and *A. sellowiana*. Total polyphenols evaluated by UPLC-PDA analysis ranged between 535.75 ± 3.11 and 635.96 ± 5.21 mg/100 g fw, and *A. sellowiana* provided the higher amount. Flavan-3-ols accounted for more than 70% of phenolic compounds, and only smoothies enriched with *C. sativus* showed a high amount of anthocyanins (25.12 ± 0.18 mg/100 g fw). The outcome of this study indicates these original smoothies as a possible ally in counteracting oxidative stress, as established by their favourable antioxidant compound profile, thus suggesting an interesting future application as nutraceuticals.

## 1. Introduction

In past years, there have been significant changes in the food industry because of the relationship between human health and nutritional food components, creating a trend for healthy eating. Consumers have started to choose products not just because of their sensory attributes but also for their nutritional and health properties, mainly due to the content of vitamins, minerals, and polyphenols. As a consequence, the food industry has started to develop and create new, healthy, safe, and attractive (in sensory terms) products to satisfy customer demand. Among ready-to-drink beverages, smoothies are conquering an increasing market interest. These are semi-liquid, smooth-consistency blended beverages, prepared by mixing fruits and vegetables or other ingredients (fruit juices, yogurt, milk, or honey) in appropriate proportions. Smoothies are regarded as so-called superfoods, defined as natural foods with beneficial and health-protecting qualities, which derive from the nutrients of the fruit components [1,2].

Products with antioxidant and/or antidiabetic properties suitable for daily use by both healthy individuals and those suffering from a wide range of chronic diseases, such as diabetes and cardiovascular diseases, are welcomed. These disorders can be caused by inappropriate diet or lifestyle, as well as the action of free radicals and reactive oxygen species (ROS) [3]. Strategies for preventing and treating these diseases include the consumption of food products naturally rich in significant amounts of antioxidant compounds, such as polyphenols and reducing vitamins [4]. Unfortunately, these compounds occurring in fresh products are partially lost in chip food, ready-to-eat foodstuffs, or highly processed products offered by food stores. Therefore, currently, an important challenge of food production technology is to guarantee the appropriate composition of beneficial human health-promoting compounds in final food products.

Phenolic compounds, such as flavonols, flavan-3-ols, phenolic acids, or anthocyanins, are an important group of natural antioxidants that are found in a variety of plants like fruits or vegetables. Moreover, they have a significant impact on our health, thanks to their biological functions such as the establishment of microbial symbioses and UV protection [5]. Polyphenols play a crucial role in preventing and reducing the progression of diabetes, cancer, and neurodegenerative and cardiovascular diseases. Furthermore, they have an important role as a prebiotic, increasing the ratio of beneficial bacteria in the gut, which is significant for health, weight management, and disease prevention. One of the best-known groups of phenolic compounds is anthocyanins, which are the reason for the plants’ red-purple colour. They have a strong antioxidant activity in metabolic reactions, based on their ability to scavenge ROS and other reactive species [6,7]. This quality makes anthocyanins a useful tool in studies on oxidative stress and its related pathologies, such as diabetes, cancer, inflammation, stroke, and Alzheimer’s disease [6,7,8,9].

Furthermore, bioactive compounds derived from fruits stimulate insulin secretion and reduce serum cholesterol, triglycerides, and blood pressure. The consumption of pectin-rich fruits, such as apples and pears, signals a low glycaemic response that determines the ability of carbohydrates to increase blood glucose in the human body [1]. Dietary carbohydrates, such as oligosaccharides and disaccharides, are hydrolysed by pancreatic α-amylase and intestinal α-glucosidase enzymes into monosaccharides suitable for absorption. The inhibition of these enzymes is particularly useful for managing non-insulin-dependent diabetes because it slows down the release of glucose in the blood. In turn, α-amylase and α-glucosidase break down polysaccharides into glucose, and pancreatic lipase breaks down triglycerides into bioavailable fatty acids and monoglyceride or glycerol molecules, so their inhibition can reduce postprandial hyperglycaemia and energy intake, respectively [10]. Therefore, the controlled reduction of enzyme activities is a promising strategy for preventing and treating type 2 diabetes mellitus jointly with a reduced dose of drugs, being overweight, and the early stages of obesity and their complications.

Following our recent research on the effect of apple juice enrichment with selected plant materials [2], this paper aimed to investigate the potential health-promoting activities of innovative smoothies obtained with strawberry tree (*Arbutus unedo,* Au) fruit puree and apple *(Malus domestica*, Md) juice enriched with *Diospyros kaki* (Dk) fruits, *Myrtus communis* (Mc) purple berry extract, *Acca sellowiana* (As), and *Crocus sativus* (Cs) petal juice. Antioxidant activity was determined by evaluating total reducing power (FRAP and CUPRAC assays), free radical scavenging activity (ABTS^•+^ and DPPH^•^ assays), and oxygen radical absorption capacity (ORAC assay). Moreover, in vitro analyses on Caco-2 cells (cytotoxic activity and determination of intracellular ROS production) were also carried out. Finally, estimation of inhibition of targeted digestive enzymes (α-amylase, α-glucosidase, and pancreatic lipase) was performed to evaluate the potential benefits for consumers with health concerns such as diabetes and weight management. Strawberry tree fruit/apple juice smoothies were investigated for their total polyphenol content by Folin-Ciocalteu’s assay, and phenolic compounds were determined in the examined samples using ultra-performance liquid chromatography photodiode detector-quadrupole time of flight/mass spectrometry (UPLC-PDA-QTof/MS) and quantified by UPLC-PDA. Furthermore, proanthocyanidin analysis and determination of sugar and organic acid content were performed.

## 2. Materials and Methods

### 2.1. Chemicals and Standards

All the chemicals used in this study were of analytical grade. Standards of phenolic compounds were purchased from Extrasynthese (Genay Cedex, France). Standards of organic acids and sugars were obtained from Merck (Darmstadt, Germany) as well as acetonitrile for ultra-pressure liquid chromatography (UPLC, gradient grade), methanol, Folin–Ciocalteu’s reagent, Trolox (6-hydroxy-2,5,7,8-tetramethylchroman-2-carboxylic acid), TPTZ (2,4,6-tripyridyl-1,3,5-triazine), DPPH^•^ (2,2-diphenyl-1-picrylhydrazyl), α-amylase from porcine pancreas (type VI-8), α-glucosidase from *Saccharomyces cerevisiae* (type I), and lipase (EC 3.1.1.3) from porcine pancreas (type II).

### 2.2. Plant Materials

All the plant material used for this experimentation was fresh and collected at commercial maturity or at the best phenological stages. *D. kaki* fruits were obtained from the plantation “Melotto” (Villacidro, Sardinia, Italy), and apples (*M. domestica* cv. Šhampion) were purchased from the LA-SAD SP. Z.O.O (Borzęcin, Błędów, Poland). Strawberry tree (*A. unedo*) fruits, myrtle *(M. communis*) berries, feijoa (*A. sellowiana*) flowers and saffron (*C. sativus*) flowers were collected with random-block design sampling by professional pickers from plants growing under environmental conditions in Sinnai, Monte Arcosu, Uta, and San Gavino Monreale (Sardinia, Italy), respectively. The specimens were identified by Prof. Andrea Maxia (University of Cagliari, Italy), and voucher samples (number DISVA.ALI.05.2021, DISVA.ALI.06.2021, DISVA.ALI.09.2021, and DISVA.ALI.10.2021) were deposited at the Department of Life and Environmental Sciences of the University of Cagliari (Italy).

### 2.3. Strawberry Tree Fruit/Apple Smoothie Production

The smoothie base (Au/Md) was prepared by mixing strawberry tree fruit mousse with apple juice (25:75, *w*/*w*). This proportion was chosen after preliminary tests, varying the amount of apple juice from 50 to 80% *w*/*w*. An amount of 75% apple juice was considered optimal due to the best semi-liquid consistency of the obtained mixtures. Strawberry tree fruit puree and apple juice were produced according to the procedure described by Gil et al. [2]. Au/Md was mixed in appropriate proportions (*w*/*w*) with other plant semi-products: 95:5 for *M. communis* berry extract (Au/Md + Mc), *D. kaki* purée (Au/Md + Dk), and *A. sellowiana* flowers (Au/Md + As), and 99.95:0.05 for *C. sativus* petal juice (Au/Md + Cs). The processing of myrtle berries, persimmon fruits, feijoa flowers, and saffron petal juice was performed according to Gil et al. [2]. Then, all products were heated to 100 °C, hot-filled in glass jars (135 mL), pasteurized (10 min at 90 °C), and cooled to 20 °C. The five different smoothies were analysed immediately after processing.

### 2.4. Determination of Total Phenolic Content, Total Reducing Power, and Free Radical Scavenging Activity

The five assays were measured spectrophotometrically in triplicate. The total polyphenolic content (TP) was estimated with a modified Folin–Ciocalteu assay [11] and the results were expressed as mg gallic acid equivalent (GAE)/100 g fw. The cupric ion-reducing antioxidant activity (CUPRAC) assay was performed according to Bektaşǒglu et al. [12] with slight modifications and the results were expressed as millimoles Fe^2+^/100 g fw. FRAP, ORAC, ABTS^•+^, and DPPH^•^ assays were performed according to the procedure previously described by Benzie and Strain [13], Ou et al. [14], Re et al. [15], and Tuberoso et al. [11], respectively, and results were expressed as millimoles of Trolox/100 g fw.

### 2.5. In Vitro Analysis on Caco-2 Cell Line

#### 2.5.1. Maintenance of Intestinal Cell Culture

Intestinal Caco-2 cells (ECACC Salisbury, Wiltshire, UK) were cultured in Dulbecco’s modified Eagle’s medium (DMEM), supplemented with 10% heat-inactivated bovine serum, 100 U/mL penicillin, 100 mg/mL streptomycin, 1% non-essential amino acids, and 2 mM L-glutamine in monolayers at 37 °C in a humidified atmosphere of 5% CO_2_ [16]. All cell culture materials were obtained from Euroclone (Pero, Italy). Caco-2 cells, at passage 45–60, were plated at a density of about 5 × 10^4^/mL and used when fully differentiated (14–21 days post seeding), replacing the medium twice a week.

#### 2.5.2. Cytotoxic Activity and Determination of Intracellular ROS Production

The MTT assay was assessed on Caco-2 cells [17] to evaluate any toxic activity of the tested extracts. The viability of cells was expressed as a percentage of cell control viability. Intracellular ROS production was evaluated in differentiated Caco-2 cells as done for the MTT assay, according to the procedure reported by Barberis et al. [18].

### 2.6. Digestive Enzyme Inhibition Assays

The α-amylase and α-glucosidase inhibitory effect of the final products was based on the Von Worthington method (1993), assayed following Nowicka et al. [19], while the inhibition of pancreatic lipase activity was determined according to Podsędek et al. [20] with slight modifications. The inhibition of these three enzymes’ activities was determined in triplicate using a UV-2401 PC spectrophotometer (Shimadzu, Kyoto, Japan) and the results were expressed as IC_50_.

### 2.7. Identification and Quantification of Polyphenolic Compounds and Analysis of Polymeric Proanthocyanidins by the Phloroglucinol Method

Polyphenols were extracted according to the procedure reported by Wojdyło et al. [21]. Briefly, in the first step, products were weighed and thoroughly mixed with 30% methanol solution, followed by the addition of acetic (1%) and ascorbic (1%) acids. The mixtures were sonicated for 15 min, stored at 4 °C for 20 h, and sonicated again for 15 min. Subsequently, all the supernatant was collected after centrifugation and the ready extracts were filtered through a 0.20 μm hydrophilic PTFE membrane (Millex Simplicity Filter; Merck, Germany). Qualitative and quantitative analyses of polyphenols were performed by LC-PDA/MS QTof and UPLC-PDA, respectively, following Wojdyło et al. [22]. For the quantitative analysis, the phenolic compounds were quantified at 280 nm (dihydrochalcones and flavan-3-ols), 320 nm (phenolic acids), 360 nm (flavonols), and 520 nm (anthocyanins). The polymeric procyanidins were analysed by the phloroglucinol method according to Kennedy and Jones [23] and the results were expressed as mg/100 g fw.

### 2.8. Determination of Sugar and Organic Acid Content

Quali-quantitative analyses of sugars and organic acids were performed by HPLC-ELSD and UPLC-PDA methods, respectively, according to the procedure reported by Nowicka et al. [24]. All determinations were performed in triplicate and the results were expressed as g/100 g fw.

### 2.9. Statistical Analysis

All data included in this study are presented as the mean value (*n* = 3) ± standard deviation. All statistical analyses were performed with Statistica version 7.0 (StatSoft, Krakow, Poland). Significant differences (*p* ≤ 0.05) between means were evaluated by a one-way ANOVA and Duncan’s multiple-range test. A correlation analysis was performed, and the evaluation of the statistical significance (*p* ≤ 0.05 and *p* ≤ 0.01) of observed differences was performed by using Spearman coefficients of correlation.

## 3. Results

### 3.1. Antioxidant Activity of Apple-Strawberry Tree Fruit Smoothies

Antioxidant activity of all products was measured by CUPRAC, FRAP, free radical-scavenging activity (DPPH^•^ and ABTS^•+^), and ORAC assays. Moreover, the TP measured by the Folin-Ciocalteu assay was evaluated in all smoothies. Results of all assays (Table 1) showed approximately the same trends among final products, with a positive Pearson correlation between TP and CUPRAC, FRAP, ABTS^•+,^ and DPPH^•^ (0.9670, 0.8161, 0.8181, and 0.6908, respectively) (Table S1a).

Among analysed final products, the highest antioxidant activity (CUPRAC method) was determined in all products in a range from 6.98 mmol Fe^2+^/100 g fw (Au/Md) to 8.75 mmol Fe^2+^/100 g fw (Au/Md + Mc). Furthermore, the addition of 5% feijoa flowers or persimmon fruits showed significantly higher antioxidant activity compared with other products in the case of ORAC and DPPH^•^ assays. Antioxidant analysis (FRAP, ORAC, DPPH^•^, and ABTS^•+^) showed that the greatest antioxidant activity has Au/Md + As (2.51, 2.12, and 1.49 mmol Trolox/100 g fw for ABTS^•+^, FRAP, and DPPH^•^, respectively) and Au/Md + Dk (5.25 mmol Trolox/100 g fw; ORAC). Greater antioxidant activity in the final products was positively associated with a significant increase in TP. In contrast, the lowest antioxidant activity was found in the pure base (Au/Md).

### 3.2. In Vitro Analysis on Caco-2 Cell Lines in Strawberry Tree/Apple Fruit Smoothies

#### 3.2.1. Cytotoxic Activity

**Figure 1:**
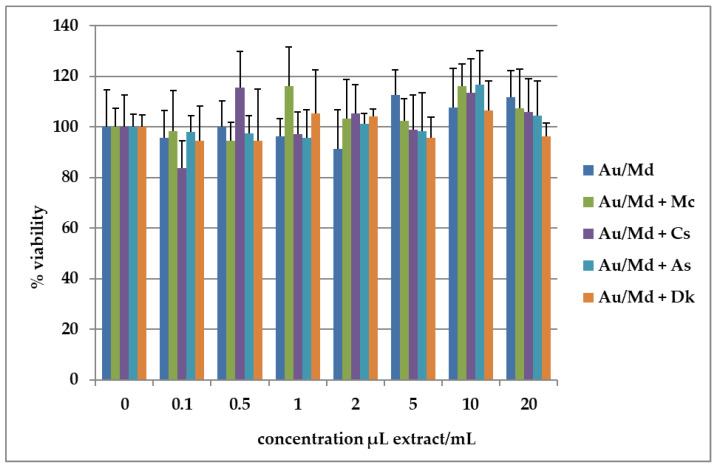
Cell viability of the best five extracts of strawberry tree fruit/apple smoothies. Results expressed as % of the control samples, measured in Caco-2 cells after incubation with different concentrations of the extracts of the five selected fresh final products or equivalent volume MeOH:H_2_O 80:20 for the controls, and incubated for 24 h. Values were shown as mean ± SD (*n* = 6 per group).

The effect of the five selected final product extracts was assessed on differentiated Caco-2 cells at concentrations ranging from 0.10 to 20 μL/mL using the MTT assay. Figure 1 summarizes the cell viability results. As it is possible to observe, the extracts of different final products did not cause a decrease in the cellular viability of Caco-2 cells, showing results of around 100% of the control (0 µL extract/mL). The statistical analysis showed that there are no significant differences (*p* > 0.05) in Caco-2 cell viability when exposed to five final product extracts with different concentrations. Despite this, analysed extracts at 0.5, 1.0, and 5.0 μL/mL showed slight differences when compared to the other concentrations under study for these extracts. Nevertheless, it should be highlighted that none of the samples caused a significant decrease in Caco-2 viability (*p* > 0.05 vs. untreated cells).

#### 3.2.2. Determination of Intracellular ROS Production

**Figure 2:**
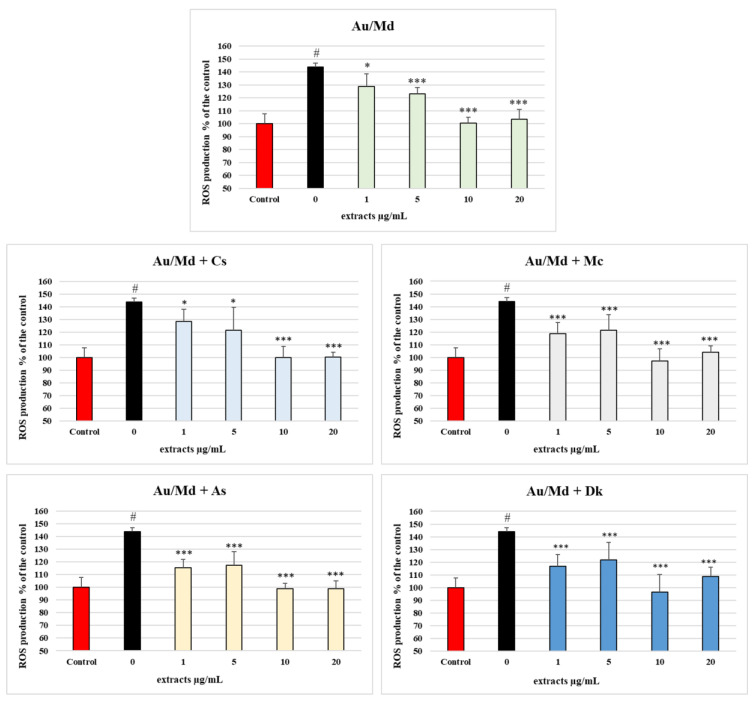
Determination of intracellular ROS production. ROS level, visualized as H2-DCF-DA fluorescence and expressed as % of the control samples (non-oxidized nor pretreated samples), in Caco-2 after 60 min incubation with TBH 2.5 mmol/L and pretreated with the extracts (0–20 µg/mL) of five selected fresh final products (Au/Md, Au/Md + Cs, Au/Md + Mc, Au/Md + As, and Au/Md + Dk) (μg/mL, 30 min). # = *p* < 0.001 vs. control; * = *p* < 0.05 vs. TBH 2.5 mmol/L; *** = *p* < 0.001 vs. TBH 2.5 mmol/L.

The ability of five selected final product extracts to scavenge reactive species was also tested in Caco-2 cell cultures (Figure 2). This human colonic epithelial cell line goes through full differentiation to enterocytes in vitro and it is extensively used to investigate the effect of nutrient components for drugs, toxicants, and contaminants, as well as for normal dietary constituents and additives [25].

Determination of intracellular ROS production and subsequent oxidative damage to cell membranes was induced by the organic hydroperoxide (TBH) [26]. In cells pretreated with all phenolic extracts, inhibition of ROS formation was observed, as indicated by the lower emission of fluorescence. Furthermore, the total phenolic concentration of tested extracts (1, 5, 10, and 20 μg/mL) showed growing, effective inhibition of ROS formation. Three tested extracts (Au/Md + Mc, Au/Md + As, and Au/Md + Dk; concentration 1 μg/mL) had slightly lower ROS levels (c.a. < 10–20%) than the other two. Moreover, the % ROS production in extracts with concentrations of 10 and 20 μg/mL was equal to the untreated cells, showing the best protective effects against oxidative damage. Overall, pretreatment with the phenolic extracts significantly attenuated the TBH-induced oxidative process in the Caco-2 cell monolayers.

### 3.3. Inhibitory Activity of Strawberry Tree Fruit/Apple Smoothies toward Digestive Enzymes 

The inhibitory activity against α-amylase, α-glucosidase, and pancreatic lipase was measured in all analysed final products. Obtained results were presented as IC_50_ values in Table 2. In general, significant differences (*p* ≤ 0.05) were found among the analysed final products in inhibitory activities toward these three digestive enzymes.

The inhibitory activity against α-amylase ranged from 64.70 (Au/Md + Mc) to 94.90 (Au/Md) mg fw/mL. The group with the strong α-amylase inhibitors includes smoothies with feijoa (As) flowers and persimmon (Dk) puree (64.83 and 65.62 mg of the final product/mL, respectively). In contrast, pure Au/Md smoothie base was characterized by the weakest α-amylase inhibition.

The values for the inhibitory activity against α-glucosidase ranged from 15.84 (Au/Md + Mc) to 27.12 (Au/Md + Cs) mg fw/mL. The strongest α-glucosidase inhibition was shown by the same group of final products as in the case of α-amylase inhibition (Au/Md + Mc, Au/Md + As, and Au/Md + Dk) and were in a range from 15.84 to 16.00 mg fw/mL. In contrast, the weakest inhibitors of α-glucosidase (26.53 and 27.12 mg fw/mL) were the pure base (Au/Md) and the smoothie with the addition of saffron (Cs) petal juice, respectively.

The inhibitory activity against pancreatic lipase ranged from 3.01 (Au/Md) to 3.14 (Au/Md + Mc) mg fw/mL and results showed moderate inhibitory activity among all products. Au/Md was the most potent inhibitor, while other products were slightly weaker (3.03 to 3.14 mg fw/mL).

### 3.4. Identification and Quantification of Phenolic Compounds in Apple-Strawberry Tree Fruit Smoothies

**Figure 3:**
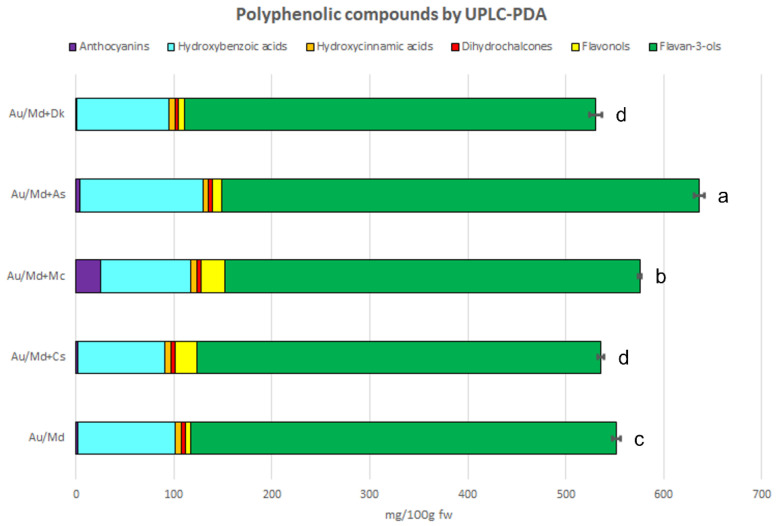
Quantification of phenolic compounds by UPLC-PDA method (mg/100 g fw) in strawberry tree fruit/apple smoothies. Data are given as mean ± standard deviation (*n* = 3). Mean values within a line with different letters (a–d) are significantly different (homogenous groups) at *p* ≤ 0.05. Total flavan-3-ols include monomers, dimers, and polymeric procyanidins.

The qualitative LC-MS analysis of the five smoothies revealed the presence of 75 different polyphenols belonging to six subclasses: anthocyanins, hydroxybenzoic and hydroxycinnamic acids, dihydrochalcones, flavan-3-ols (monomers, dimers, trimer, and polymeric procyanidins) and flavonols (Figure 3, Table S2). These compounds varied between the analysed product, and LC-MS metabolic profiles of Au/Md, Au/Md + Cs, Au/Md + Mc, Au/Md + As, and Md-AU + Dk highlighted the presence of 37, 44, 45, 48, and 42 phenolic compounds, respectively.

Besides the high amount of polymeric procyanidins and hydroxybenzoic acids in all smoothies, anthocyanins (25.12 mg/100 g fw) and flavonols (25.03 mg/100 g fw) dominated in the Au/Md + Mc smoothie, while in the Au/Md + Cs smoothie, flavonols (22.59 mg/100 g fw) additionally dominated.

The quantitative analysis showed statistically significant differences (*p* ≤ 0.05) between the obtained results of all analysed final products. The sum of polyphenols (Figure 3, Table S2) evaluated by UPLC-PDA analysis varied in all investigated smoothies and, depending on the type of plant material additive, the total quantity of detected polyphenols in final products varied (535.75–635.96 mg/100 g fw). Supplementation with 5% purple myrtle berry extract (575.86 mg/100 g fw) or feijoa flowers (635.96 mg/100 fw) significantly enriched the final products in natural antioxidants.

The positive mode LC-MS analysis highlighted the presence of a total of nine anthocyanins, and the total amount ranged between 530.72 and 635.96 mg/100 g fw (Table S2). The pure Au/Md smoothie was rich in these molecules, mainly cyanidin-3-*O*-galactoside and cyanidin-3-*O*-arabinoside. The smoothie with the addition of 5% purple myrtle (Mc) berry extract was the richest in anthocyanins (25.12 mg/100 g fw), and was enriched in delphinidin-3-*O*-galactoside and -3-*O*-glucoside, peonidin-3-*O*-glucoside, and malvidin-3-*O*-glucoside. These four molecules were only present in the smoothies enriched in the myrtle semi-product. In addition, malvidin-3-*O*-glucoside and delphinidin-3-*O*-glucoside were the molecules detected in the highest amount (10.47 and 8.03 mg/100 g fw, respectively) among all anthocyanins. Moreover, only Mc- and Cs-enriched products contained petunidin-3-*O*-glucoside (0.65 and 0.03 mg/100 g fw, respectively). The addition of saffron petal juice gave uniqueness to the final product, which contained delphinidin-3,5-*O*-diglucoside (0.35 mg/100 g fw). Regarding the anthocyanin profile, it was observed that all products were rich in cyanidin-3-*O*-galactoside in the range of 1.04 to 3.16 mg/100 g fw. Furthermore, with myrtle semi-product addition, the base was enriched with cyanidin-3-*O*-galactoside by almost double (3.16 mg/100 g fw).

The negative mode LC-MS analysis highlighted in the investigated smoothies the presence of a total of 24 hydroxybenzoic acids and their derivatives, 5 hydroxycinnamic acids, 2 dihydrochalcones, 6 flavan-3-ols, and 29 flavonols (Table S2). All hydroxycinnamic acids, dihydrochalcones, and flavan-3-ols were detected in significantly different quantities in all products.

Regarding the composition of hydroxybenzoic acids and their derivatives, interesting differences were observed. All smoothies contained gallic acid glucoside I and II, galloyl glucoside I, theogallin, galloyl shikimic acid, digalloylquinic acid I, and digalloyl shikimic acid I. Furthermore, galloyl glucoside II (2.01 mg/100 g fw) and salicylic acid (0.08 mg/100 g fw) were detected only in Au/Md + Dk, while castalagin (4.26 mg/100 g fw), casuarina (1.69 mg/100 g fw), ellagitannin I (15.28 mg/100 g fw), digalloylquinic acid II (2.26 mg/100 g fw), ellagitannin III (0.71 mg/100 g fw), nilocitin (0.41 mg/100 g fw), and casuarinin (0.28 mg/100 g fw) were detected only in Au/Md + As. Au/Md + Mc was the only product that contained quinic acid 3,5-di-*O*-gallate and ellagitannin II (0.31 and 0.11 mg/100 g fw, respectively), while Au/Md + Cs was the only product rich in gallotannin derivative (B20; 0.43 mg/100 g fw). Gallic acid 4-*O*-β-D-glucopyranoside was detected in all final products (0.08–0.41 mg/100 g fw) except product Au/Md + As. Moreover, digalloyl shikimic acid II (3.02–3.39 mg/100 g fw) was detected in all products except Au/Md + Mc and Au/Md + As, while ellagic acid arabinoside (0.92–4.81 mg/100 g fw) was found in all products except Au/Md + Cs. Interestingly, ellagic acid xyloside (0.88 and 0.74 mg/100 g fw) and ellagic acid (4.30 and 0.81 mg/100 g fw) were detected only in Au/Md + As and Au/Md + Dk, respectively.

Among the detected hydroxycinnamic acids were neochlorogenic and chlorogenic acid, *p*-coumaroyloquinic acid, caffeic acid, and *p*-coumaric acid. The total hydroxycinnamic acid content in all products ranged between 5.65 and 6.16 mg/100 g fw. Moreover, it was noticed that each additional component decreased the amount of these compounds, except for feijoa (As) flowers. Chlorogenic acid was the major representative of this group of polyphenols (4.27–4.57 mg/100 g fw), while four other acids were detected in much lower quantities (0.08–0.77 mg/100 g fw).

Two dihydrochalcones (phloretin-2′-*O*-xyloglucoside and phloretin-2′-*O*-glucoside) were present in all smoothies, probably solely from apples. The total amount ranged between 3.47 and 4.06 mg/100 g fw. It was noticed that the addition of saffron (Cs) petal juice and myrtle (Mc) berry extract enriched the end product in these polyphenols, while the addition of feijoa flowers and persimmon puree decreased the total amount of dihydrochalcones. Phloretin-2’-*O*-glucoside was present in significantly higher amounts (2.03–2.22 mg/100 g fw) in all products, followed by phloretin-2’-*O*-xyloglucoside (1.42–1.87 mg/100 g fw).

In the case of flavan-3-ols, procyanidin B1, B2, B3, and C1, as well as (+)-catechin and (−)-epicatechin were present in all smoothies. The total content of these compounds ranged between 412.44 and 487.37 mg/100 g fw (Table S2). The greatest quantity of flavan-3-ols was detected in products enriched in 5% feijoa flowers, while the lowest was in product with added 0.5% saffron petal juice. A reduced amount of flavan-3-ols was observed while adding the semi-products, except Au/Md + As. Regarding each particular compound, it was observed that procyanidin B1 (2.20–19.36 mg/100 g fw) and two dimmers (10.14–13.03 and 1.05–6.83 mg/100 g fw) were the major flavan-3-ols. Interestingly, myrtle berry extract increased the amount of procyanidin B1 by three times, but decreased the amount of (−)-epicatechin by c.a. six times. On the other hand, feijoa (As) flowers and persimmon (Dk) puree addition decreased procyanidin B1 content by three and two times, respectively. Furthermore, polymeric procyanidins were detected in the highest amount in Au/Md + As, and their total amount in all analysed products ranged between 384.42 and 463.40 mg/100 g fw.

The total amount of flavonols in the analysed smoothies ranged between 5.90 and 25.03 mg/100 g fw. Au/Md + Cs and Au/Md + Mc were the products richest in flavonols. Three myricetin-3-*O* derivatives were found in all smoothies, as follows: galactoside (0.11–9.32 mg/100 g fw), xyloside (0.24–032 mg/100 g fw), and rhamnoside (0.12–5.78 mg/100 g fw). Two quercetin-3-*O*-hexosides were found in all smoothies, as follows: galactoside (0.88–1.65 mg/100 g fw) and glucoside (0.23–0.49 mg/100 g fw). Furthermore, analysis by LC-MS confirmed in all smoothies the presence of quercetin-3-*O*-rhamnoside (1.52–2.54 mg/100 g fw) and two quercetin-*O*-pentosides, as follows: arabinoside (0.11–0.27 mg/100 g fw) and xyloside (1.29–1.43 mg/100 g fw).

Unique to juices enriched with saffron petal juice were seven other flavonols. The mass spectrometric characterization and UPLC-PDA quantification of compounds provided evidence for the presence of three kaempferol derivatives: kaempferol-3-*O*-sophoroside-7-*O*-glucoside, kaempferol-3,7-*O*-diglucoside, and kaempferol-3-*O*-sophoroside (0.34, 0.12, and 9.53 mg/100 g fw, respectively) in Au/Md + Cs. Another four were detected as isorhamnetin derivatives: isorhamnetin-3,7-*O*-digalactoside, isorhamnetin-3,7-*O*-diglucoside, isorhamnetin-3-*O*-rutinoside, and isorhamnetin-3-*O*-glucoside (0.75, 1.66, 1.01, and 0.07 mg/100 g fw, respectively). Moreover, quercetin-3,7-*O*-diglucosidase was found in Au/Md + Cs and Au/Md + As smoothies, in amounts of 1.80 and 0.10 mg/100 g fw, respectively. Feijoa flowers provided a unique flavonol profile to the final product. The following were found only in Au/Md + As: kaempherol-3-*O*-galactoside (0.55 mg/100 g fw), kaempferol-hexoside (0.15 mg/100 g fw), and quercetin and kaempferol (0.12 and 1.80 mg/100 g fw, respectively). Additionally, both As and Dk enriched the final product in quercetin-pentoside (0.50 and 0.11 mg/100 g fw, respectively). Also, myrtle berry extract gave a unique flavonol profile to the final products. Mc enriched smoothies in myricetin galactoside-gallate, myricetin-3-*O*-arabinoside, and myricetin (1.35, 1.06, and 0.36 mg/100 g fw, respectively). Other flavonols, such as quercetin derivative I, were found in all smoothies (0.30–0.46 mg/100 g fw) except Au/Md + Cs, or both myricetin-3-*O*-glucoside and quercetin galloylhexoside were detected in all products (0.04–0.86 and 0.26–0.51 mg/100 g fw, respectively) except Au/Md + As.

### 3.5. Sugar and Organic Acid Content of Apple-Strawberry Tree Fruit Smoothies

Table 3 reports the sugar and organic acid content, and shows that mixing various plant materials into the strawberry tree fruit/apple smoothie allowed for enriching the new smoothies with specific sugars and organic acids (enriched products differed significantly from the Au/Md smoothie, *p* ≤ 0.05). Some significant Spearman correlations were also observed (Table S1b).

For the sugar and organic acid content, the standard mixture of selected sugars and organic acids, respectively, was prepared. The exact profile of these compounds in analysed final products was reported according to their retention time (Rt) order and the total content of these compounds.

Generally, fructose, glucose, sucrose, and sorbitol were detected in all final products. The total sugar content in all final product processing ranged from 13.27 to 16.50 g/100 g fw. The highest total sugar content was found in the smoothie with myrtle purple berry extract, followed by the smoothie with feijoa flowers and saffron petal juice (15.87 and 15.36 g/100 g fw, respectively). In contrast, the lowest total content of sugar was detected in the pure base, followed by the smoothie with persimmon puree (13.34 g/100 g fw).

Fructose and glucose were the most abundant sugars, while the sorbitol and sucrose contents were much lower. However, after analysing every single sugar, some interesting results were observed. A similar trend to total sugars was observed in the case of fructose, whose content ranged from 10.11 to 13.49 g/100 g fw. The highest fructose content was detected in product Au/Md + Mc, followed by products Au/Md + As and Au/Md + Cs (12.37 and 12.39 g/100 g fw, respectively), while the lowest fructose content was found in the pure base (Au/Md).

On the other hand, the glucose content ranged from 2.47 to 3.19 g/100 g fw. Here, the situation was different than in the case of fructose. It was observed that the addition of the other semi-products decreased the general amount of glucose in all prepared smoothies except the one with feijoa flowers, the richest in this sugar. In turn, the lowest content of glucose was observed in the smoothie containing an additional component of persimmon purée (2.47 g/100 g fw). Finally, sorbitol and sucrose content in all smoothies ranged from 0.03 to 0.09 and 0.22 to 0.31 g/100 g fw, respectively. The highest content of sorbitol was detected in product Au/Md + Cs, while the lowest was in product Au/Md + Mc. Regarding sucrose, the highest content was evaluated in product Au/Md + Mc, while the lowest was in both products Au/Md + As and Au/Md + Dk.

Eight organic acids were identified and quantified in analysed final products: oxalic, citric, isocitric, malic, quinic, ascorbic, shikimic, and fumaric acid. The study showed that the organic acid content of analysed beverages differed significantly (*p* ≤ 0.05). Generally, the total organic acid content in all analysed smoothies ranged from 2.12 to 3.09 g/100 g fw. The highest total organic acid content was found in products with feijoa flowers, while the lowest was in smoothies with saffron petal juice.

Detailed analysis of organic acid content in analysed smoothies showed that quinic and malic acid were the most abundant organic acids among all, while other acids were detected in much lower quantities or not detected at all. After analysing every single organic acid, some interesting results were observed. Quinic and malic acid were detected in smoothies in ranges from 1.41 to 1.76 g/100 g fw and 0.63 to 0.75 g/100 g fw, respectively. The highest content of quinic acid was found in Au/Md + As, while the lowest was observed in Au/Md + Cs. In the case of malic acid, the same trend was observed.

On the other hand, oxalic and citric acids were detected in all final products in much lower quantities (0.03–0.19 and 0.07–0.20 g/100 g fw, respectively) than the other two acids. Isocitric acid was specific to some final products (Au/Md, Au/Md + Mc, and Au/Md + As). The amount of this acid present in these three products was 0.39, 0.25, and 0.31 g/100 g fw, respectively. Finally, the presence of ascorbic, shikimic, and fumaric acids was confirmed in all final products. The quantity of these three acids was very low (≤0.01 or traces).

Last but not least, the total sugar/organic acid ratio was investigated in all analysed smoothies. It was noticed that each additional component (Cs, Mc, As, and Dk) increased the general sugar: organic acid ratio. The ratio for the Au/Md smoothie was 4.88, while other product ratios ranged between 5.14 and 6.77.

## 4. Discussion

The present study aimed to evaluate the antioxidant activity and inhibition of targeted digestive enzymes of five original smoothies, in relation to their polyphenolic and organic acid composition deriving from the addition of some fruits and flowers in a strawberry tree fruit and apple juice base. Regarding the scavenging properties of the five smoothies tested in this investigation, we first observed a positive correlation between TP and antioxidant activity (Spearman correlation: 0.9670, 0.8161, 0.6908, and 0.8181 for CUPRAC, FRAP, DPPH^•^, and ABTS^•+^, respectively) (Table S1a). These polyphenol contents in the analysed smoothies partially confirmed the data obtained by HPLC-PDA analysis. Also, different authors [27,28] confirmed the strong antioxidant properties of the polyphenols, and the significant correlation between phenolic concentration and free radical scavenging activity. Differences in molecular structure, including the number or placement of binding hydroxyl groups, may result in different antioxidant activity. According to Ou et al. [14], antioxidant activity is determined not only by the total content of polyphenols, but also by their chemical subclass composition. For instance, as suggested by Wojdyło et al. [29] and Nowicka et al. [1], antioxidant potential depends on the presence of anthocyanins, flavonols, and polymeric procyanidins. The results presented in this study showed that the antioxidant activity of the analysed smoothies correlated well with the total hydroxybenzoic acid and its derivatives, dihydrochalcones, flavan-3-ols, and polymeric proanthocyanidins (Pearson correlation for DPPH^•^: 0.8091, 0.9059, 0.8028, and 0.8162, respectively; Pearson correlation for ABTS^•+^: 0.8133, 0.7797, 0.8399, and 0.7883, respectively; Pearson correlation for FRAP: 0.7572, 0.8818, 0.7609, and 0.7548, respectively) (Table S1a). The correlations with the above-mentioned groups of polyphenols and other antioxidant assays were instead significantly much lower. Given all these results, we therefore decided to test the phenolic extracts deriving from smoothies in in vitro biological systems as intestinal cell cultures.

Before testing direct antioxidant activity, the five phenolic extracts deriving from our freshly prepared smoothies were tested on differentiated Caco-2 intestinal cells to ascertain the absence of toxicity at increasing concentrations (0–20 µL extract/mL medium) by the routine MTT test. According to the findings of De Francisco et al. [30], Caco-2 cell lines are often used as intestinal models to investigate the effect of novel food ingredients on cell viability. Moreover, as described by Meunier et al. [31], the functional characteristics and morphology of Caco-2, when used as differentiated cells, are very like enterocytes (tight junctions and apical and basolateral layers, as well as microvilli present on the apical surface). In our experiments, cell viability remained unchanged in the presence of the Au/Md extract at all concentrations tested, compared to untreated cells (100% viability). Notably, the same outcome occurred for cells treated with Au/Md smoothie supplemented with fruits (Mc, Dk, As, and Cs). The addition of phenolic substances to the already concentrated Au/Md extract (as shown in Table 1) did not lead to an increase in toxicity in Caco-2 monolayers, therefore it was possible to further test these concentrations to evaluate the antioxidant activity of the five extracts.

The capacity of phenolic compounds coming from smoothies to exert a notable antioxidant activity was then tested in the same experimental model. The potential protective effect of the extracts against TBH-induced oxidative damage in Caco-2 cell monolayers was investigated (Figure 2). As expected, a highly significant increase in ROS production was caused by TBH, in comparison with the untreated cells (Control). In the presence of the phenolic extracts coming from the Au/Md smoothie, ROS production significantly decreased starting from 1 µg/mL (*p* < 0.05) in comparison with the positive control (TBH 2.5 mM alone), and it lowered as the smoothie extract concentrations grew; at 10 and 20 µg/mL, the highest concentration tested, the extract was even able to increase ROS production to a level similar to the untreated cells, completely nullifying the effects of the TBH incubation. Similar results were also observed in the extracts of the smoothies supplemented with the different flowers and fruits which were the objects of our investigation. Indeed, as expected, some of these (Au/Md + Mc, Au/Md + As, and Au/Md + Dk) showed a more significant ROS decrease (*p* < 0.001) already at the lowest concentration tested (1 µg/mL), probably due to a higher concentration of TP as reported in Table 1. This finding confirms that the addition of some of the tested fruits increased the antioxidant capacity of the Au/Md smoothie even in a biological system such as the Caco-2 cell monolayer, confirming what was seen in the other antioxidant capacity tests. This protective action of the smoothies’ phenolic extract is likely due to the ability of its main components to directly scavenge TBH-generated radicals or intermediates of reaction metabolites [26].

These results are of relevance, since polyphenols, once ingested with the diet, easily concentrate in the gut; thus, they are able to exert their biological function against the prooxidant agents deriving from metabolism or ingested with the diet (e.g., oxidized lipids) [16]. Therefore, the smoothies studied in this research could represent useful tools for the intake of dietary bioactive compounds.

Synchronous and strong inhibition of α-amylase and α-glucosidase can lead to disturbances of the gastrointestinal system because of the presence of undigested carbohydrates in the colon, leading to negative bacterial fermentation. Therefore, a strong inhibition of one of the enzymes and moderate inhibition of the other is desirable [32]. It is worth noting that the presence of *D. kaki* and *A. unedo* fruits in products has a positive influence on digestive enzyme inhibition. According to Wang et al. [33], inhibition of α-glucosidase is associated with the content of hydroxycinnamic derivatives, such as *p*-coumaric or ferulic acids, but in this study and in the study of Nowicka et al. [1], no correlation was found between this subclass of phenolic acids and α-amylase, α-glucosidase, and pancreatic lipase. This suggests that other polyphenols (anthocyanins, flavonols, or polymeric procyanidins) might contribute to the inhibitory effect on digestive enzymes. Indeed, thanks to different mechanisms, most of the detected phenolic compounds were reported to have an antidiabetic effect. It is well established that big differences in structure among different subclasses of polyphenols and within the group affect their solubility, stability, and bonding ability with the digestive enzymes [34]. Akkarachiyasit et al. [35] claimed that cyanidin-3-*O*-galactose was a very good inhibitor of rat intestinal sucrose activity (α-glucosidase inhibition). Boath et al. [36] suggested that flavonols may interact with phenolic acids or anthocyanins, causing the inhibition of α-glucosidase. Furthermore, Liu et al. [37] highlighted the importance of the ellagitannin derivatives, such as punicalagin, in managing type 2 diabetes. In this study, the Authors conclude that the inhibitory activity of punicalagin against α-glucosidase can be due to both competitive and uncompetitive inhibition, probably via directly binding with α-glucosidase [37]. These findings may be an additional explanation for why the investigated product containing feijoa flowers (Au/Md + As) had a strong inhibitory effect on *α*-glucosidase; this functional beverage was rich in different ellagic acid derivates that might be responsible for the enzyme inhibition.

α-Glucosidase and pancreatic α-amylase and lipase may be effective in the regulation of hyperglycaemia and type 2 diabetes by controlling glucose absorption [20,38]. Moreover, as documented by Picot et al. [39], other compounds, like fibres or viscous polysaccharides, can contribute to the inhibition of digestive enzymes due to decreases in the postprandial plasma glucose level. Finally, it was observed that after mixing the components of bases (apple juice and strawberry tree fruits) and other plant semi-products, polyphenols of the base smoothie interacted with polyphenols contained in other plant materials, and as a consequence showed high anti-α-amylase and anti-α-glucosidase activities, thereby creating a highly valuable final product with the potential to lower the risk of obesity and diabetes.

Mixing plant materials with different polyphenolic profiles leads to diversity in chemical composition, which guarantees specific biological advantages and the high quality and nutrition of the final product [40]. Moreover, it has been widely observed that polyphenols pertaining to different subclasses exert antioxidant effects with different mechanisms. Some, for example, are able to chelate metals that catalyse the formation of radical species [41]; others are able to exert a pure scavenging action and to interrupt the free radical chain reactions which are typical, for example, of lipid peroxidation [42,43]. All these mechanisms may contribute to the overall antioxidant action of the polyphenol-rich food matrices, showing a synergistic action.

According to Kschonsek et al. [44], generally, five major polyphenolic groups are found in various apple varieties: flavonols (quercetin and isorhamnetin derivatives), hydroxycinnamic acids, flavan-3-ols (catechin, epicatechin, and procyanidins), anthocyanins (mainly cyanidin derivatives), and dihydrochalcones (phloretin glucosides).

The strawberry tree fruits are rich in different phenolic acids, such as gallic, gentisic, protocatechuic, and *p*-hydroxybenzoic [45], and other phenolic compounds: anthocyanins (cyanidin-3-*O*-β-D-galactopyranoside, delphinidin-3-*O*-β-D-glucopyranoside, and cyanidin-3-*O*-β-D-arabinoside) [46], proanthocyanidins (>80% of the total flavonoid content), quercetin, kaempferol, naringin, catechin, apigenin, and others [47].

In recent years, saffron floral by-products obtained after stigma separation have been investigated as potential sources of bioactive compounds [48,49]. The major phenolic compounds investigated in *C. sativus* flower juice are flavonols, such as kaempferol-3-*O*-sophoroside and other kaempferol derivatives, quercetin, and isorhamnetin glycosides. Anthocyanins present in saffron flower juice are mainly delphinidin-3,5-di-*O*-glucoside followed by delphinidin 3-*O*-glucoside, malvidin 3,5-di-*O*-glucoside, petunidin 3-*O*-glucoside, and petunidin-3,7-di-*O*-glucoside.

Feijoa flowers (buds) were studied for the first time regarding their content in bioactive compounds and health properties by Aoyama et al. [50] and Montoro et al. [51]. Polyphenols investigated and present in feijoa flower bud extract are ellagic and gallic acid, representative of tannins—pedunculagin, flavone, and an anthocyanin constituent, cyanidin glucoside.

Purple myrtle berries have been widely investigated for their chemical composition and they are rich in tannins, phenols, fatty acids, and essential oils. The phenolic compound fraction contains gallic acid, flavonoids, and anthocyanins. Anthocyanins are the most abundant phytochemicals in myrtle berries, represented by five anthocyanin glucosides and four anthocyanin arabinosides, with malvidin-, delphinidin-, and cyanidin-3-*O*-glucoside as major compounds. The flavonol profile was characterized by myricetin-3-*O*-galactoside, myricetin-3-*O*-rhamnoside, and aglycon of myricetin and quercetin [52,53].

Persimmon fruits are the source of many bioactive compounds, such as polyphenols, vitamins (vitamins B1, B2, B3, A, E, K, and C), minerals (calcium and potassium), sugars (sucrose and its glucose and fructose monomers), carotenoids, tocopherols [54], and dietary fibres [55]. Generally, the main polyphenols in these fruits are flavonols (quercetin and rutin), hydroxycinnamic acids (caffeic, *p*-coumaric, and ferulic acid), hydroxybenzoic acids (gallic, vanillic, and syringic acid), and flavan-3-ols ((+)-catechin) [56].

In general, the above scientific findings regarding polyphenols present in the studied plant materials were confirmed by analyzing the enriched strawberry tree fruit/apple smoothies. Interestingly, the addition of each plant material increased the phenolic composition of functional smoothies. It is also noteworthy that the final products were rich in fructose, which has better metabolic properties than glucose and sucrose due to its lower glycaemic index. Furthermore, fructose and glucose are sweeter than sucrose [22], and fructose has higher relative sweetness than glucose [57]. Also, the presence of sorbitol can have a positive effect, because it is non-cariogenic; thus, it can protect against caries (tooth decay). Moreover, it slows the rise of blood glucose and the insulin response connected to the ingestion of glucose. For this reason, it is used as a sugar alternative for people with diabetes [58]. We assume in this investigation that the presence of fructose and glucose in analysed smoothies may guarantee the sweetness of beverages, and these beverages will not require any additional sweeteners. Moreover, fructose was generally found to be more abundant than glucose in investigated smoothies. Fructose, which is one of the most important dietary monosaccharides, is known to be the sweetest of all naturally occurring carbohydrates [59]. Moreover, taking into account that fructose has a lower glycaemic index and higher sweetness index than glucose [60], it does not lead to a rapid rise in blood glucose levels.

On the other hand, the presence of particular organic acids guarantees specific tastes, flavours, and aromas, as well as plays a role in stabilizing and preserving foodstuffs [61]. The appearance of different organic acids exerts a significant influence on a fruit’s taste and colour (depending on pH), also taking into account the sugar/acid ratio [62,63]. Recent studies [1,2] have shown that, by mixing different fruits, final products with specific qualities can be designed. Therefore, the organic acid profile can be influenced by the ratios in which different fruits are mixed and used for final product preparation.

Each organic acid present in our smoothies possesses specific health benefits. These compounds stimulate the secretion of digestive enzymes, as well as regulate the chemical reactions of the body [64]. Malic acid is the predominant organic acid in apple fruits and plays a role in maintaining healthy liver conditions and helping in the digestion process [65]. Quinic acid is a principal biochemical intermediate in the shikimate pathway (of aromatic compounds occurring in plants, bacteria, fungi, and algae) but it is not biosynthesized in animals and humans [66]. Citric acid, aside from being a natural preservative, gives foods and beverages a sour taste. Moreover, it is a metabolite and an intermediary in the oxidative metabolism of cells [67]. Oxalic acid is a final product in the metabolism of some amino acids in mammals, and dietary intake is c.a. 50 mg, depending on the type of food [68]. According to the findings of Iqbal et al. [69], ascorbic acid is an antioxidant that protects humans from oxidative stress damage. This compound is crucial in wound healing, bone formation, and the preservation of healthy gums. Moreover, this acid has an important role in various metabolic functions (e.g., the activation of vitamins from group B and folic acid, alteration of cholesterol to bile acids, and alteration of amino acids to serotonin—neurotransmitters). Therefore, the dietary intake of these organic acids with our smoothies could be important in terms of nutrition, health, and well-being in humans because of their wide range of beneficial functions.

Different scientific research studies have noticed the relationship between the sugar: organic acid ratio and consumer desirability. Jaros et al. [70] observed that, when considering cloudy juices, the most desired products had a low sugar: organic acid ratio. However, the research indicated that there is an optimum for this ratio. Therefore, this scientific group pointed out that in the range limited by samples tested, preference may be rising with the increasing sugar: organic acid ratio. Also, in work by Tkacz et al. [71], the authors confirmed the significant influence on sensory qualities when increasing the sugar: organic acid ratio, which improves the attractiveness of taste, colour, and aroma. The above scientific findings and results presented in this study may indicate that the smoothies enriched with Cs, Mc, As, and Dk may be more attractive to the consumer than the pure base (Au/Md).

This work aimed to develop an innovative recipe concept based on apple fruits, which are known and commonly used in the food industry, as well as strawberry tree fruits, which are rarely applied in food products thus far. Strawberry tree fruits, despite their high nutritional value, are generally used to produce highly processed products with a low Nutri-Score. These products can be highly sweetened jams, jellies, marmalades, or alcoholic drinks (liqueurs, wines, and brandies) [72], and they do not fit in the direction of novel food design with health-promoting characteristics.

For the first time, the authors proposed an innovative product with health-promoting potential using strawberry tree fruits. In their opinion, this new product in the form of a smoothie has a high application value. The product is characterized by attractive sensory qualities, so it may gain recognition among consumers. It is worth emphasizing that so far these fruits have not been used for processing purposes, and require minimal agrotechnical treatments during the ripening process. Therefore, from an economic point of view, this type of product will require a small amount of money and will fit into sustainable development.

Besides the promising base, another important aspect of the innovation was the development of the new functional products by fortification of the mentioned base with selected plant semi-products (*Diospyros kaki* fruits, *Myrtus communis* purple berry extract, *Acca sellowiana*, and *Crocus sativus* petal juice). The addition of individual semi-products affected the health value of the formulas obtained. As an example, *M. communis* enriched the base significantly with a range of different anthocyanins, providing a pleasant colour that could be attractive to the consumer. Furthermore, its addition improved the general antioxidant and antidiabetic activity of the product.

Enriching the base with these particular raw materials will require more financial outlay, but will result in the development of the product in a premium category with defined health-promoting properties. In the application context, these types of products have added value and fit into the global trend of designing healthy and functional drinks.

These are preliminary studies that allowed us to evaluate some biological activities of the smoothies under investigation. Since these studies were carried out in in vitro models, they show several limits of translatability in vivo; therefore, subsequent studies will be necessary in animal models and possibly in observational and/or interventional clinical studies to assess with greater precision the beneficial effects related to the consumption of these nutraceutical preparations.

## 5. Conclusions

In the present research, new smoothies obtained from some typical Mediterranean plant products were investigated and valorised for their chemical composition. Results showed that a strawberry tree/apple fruit smoothie is an interesting source of bioactive compounds with antioxidant activity and inhibitory action on some digestive enzymes. Enrichment of this base smoothie with different plant materials, such as *M. communis* berries, *D. kaki* fruits, *A. sellowiana flowers*, and *C. sativus* petals generally increased the nutraceutical potential of the base smoothie, especially when using the first three plant materials. The use of these plant materials is recognized to be of added value due to the marked territorial identity and biodiversity preservation. The exploitation of these smoothies may therefore support the local food production chain, and at the same time help to promote adequate lifestyles, favouring the development of a new therapeutic and preventive approach.

## Figures and Tables

**Table 1:** Antioxidant activity of analysed apple-strawberry tree fruit smoothies.

Parameter	Sample				
	Au/Md	Au/Md + Cs	Au/Md + Mc	Au/Md + As	Au/Md + Dk
TP ^†^	247.97 ± 1.49 c	258.63 ± 5.75 b	288.15 ± 3.13 a	285.68 ± 9.22 a	276.17 ± 9.73 a
CUPRAC ^‡^	6.98 ± 0.26 d	7.18 ± 0.73 cd	8.75 ± 0.07 a	8.20 ± 0.64 bc	8.17 ± 0.65 bc
FRAP ^‡^	1.95 ± 0.05 b	1.97 ± 0.05 ab	2.02 ± 0.02 ab	2.12 ± 0.10 a	2.04 ± 0.01 a
ORAC ^‡^	4.21 ± 0.21 b	3.88 ± 0.06 c	4.22 ± 0.25 b	4.49 ± 0.13 b	5.25 ± 0.27 a
DPPH ^‡^	1.25 ± 0.02 d	1.16 ± 0.05 e	1.30 ± 0.02 c	1.49 ± 0.01 a	1.37 ± 0.04 b
ABTS^•+ ‡^	2.07 ± 0.02 d	2.06 ± 0.03 d	2.28 ± 0.05 b	2.51 ± 0.01 a	2.19 ± 0.05 c

^†^ mg GAE/100 g fw; ^‡^ mmol Trolox/100 g fw; Data are given as mean ± standard deviation (*n* = 3). Mean values within a line with different letters (a–e) are significantly different (homogenous groups) at *p* ≤ 0.05.

**Table 2:** Digestive enzyme inhibitory activities (IC_50_, mg fw/mL) of analysed strawberry tree/apple fruit smoothies.

Enzyme Inhibition	Sample				
	Au/Md	Au/Md + Cs	Au/Md + Mc	Au/Md + As	Au/Md + Dk
*α*-amylase	94.90 ± 0.06 d	79.92 ± 0.10 c	64.70 ± 0.01 a	64.83 ± 0.01 a	65.62 ± 0.08 b
*α*-glucosidase	26.53 ± 0.17 c	27.12 ± 0.06 d	15.84 ± 0.04 a	15.86 ± 0.03 a	16.00 ± 0.00 b
pancreatic lipase	3.01 ± 0.06 a	3.03 ± 0.08 ab	3.14 ± 0.03 b	3.09 ± 0.11 ab	3.11 ± 0.08 ab

Data are given as mean ± standard deviation (*n* = 3). Mean values within a line with different letters (a–e) are significantly different (homogenous groups) at *p* ≤ 0.05.

**Table 3:** Sugar and organic acid (g/100 g fw) content in strawberry tree fruit/apple smoothies.

Parameter	Sample				
	Au/Md	Au/Md + Cs	Au/Md + Mc	Au/Md + As	Au/Md + Dk
Sugar content					
Fructose	10.11 ± 0.12 d	12.39 ± 0.06 b	13.49 ± 0.05 a	12.37 ± 0.00 b	10.60 ± 0.02 c
Sorbitol	0.04 ± 0.00 d	0.09 ± 0.00 a	0.03 ± 0.00 e	0.08 ± 0.00 b	0.05 ± 0.00 c
Glucose	2.86 ± 0.00 b	2.64 ± 0.01 c	2.67 ± 0.22 bcd	3.19 ± 0.19 a	2.47 ± 0.04 d
Sucrose	0.26 ± 0.01 b	0.24 ± 0.01 b	0.31 ± 0.02 a	0.22 ± 0.00 c	0.22 ± 0.00 c
Total	13.27 ± 0.13 d	15.36 ± 0.08 c	16.50 ± 0.29 a	15.87 ± 0.19 b	13.34 ± 0.06 d
Organic acid content					
Oxalic	0.04 ± 0.00 b	0.03 ± 0.00 c	0.04 ± 0.01 bc	0.19 ± 0.01 a	0.04 ± 0.00 ab
Citric	tr	0.13 ± 0.03 b	0.07 ± 0.01 c	0.08 ± 0.01 c	0.20 ± 0.03 d
Isocitric	0.39 ± 0.02 a	nd	0.25 ± 0.02 c	0.31 ± 0.02 b	nd
Malic	0.71 ± 0.03 ab	0.63 ± 0.06 b	0.70 ± 0.02 ab	0.75 ± 0.03 a	0.70 ± 0.01 b
Quinic	1.57 ± 0.00 b	1.47 ± 0.03 c	1.64 ± 0.12 ab	1.76 ± 0.06 a	1.54 ± 0.05 bc
Ascorbic	tr	tr	tr	tr	tr
Shikimic	tr	tr	tr	tr	0.01 ± 0.00 a
Fumaric	tr	tr	tr	tr	tr
Total	2.72 ± 0.05 b	2.27 ± 0.12 c	2.70 ± 0.18 b	3.09 ± 0.13 a	2.50 ± 0.09 bc
Sugars/organic acids	4.88 ± 0.14 a	6.77 ± 0.41 b	6.11 ± 0.50 ab	5.14 ± 0.28 a	5.34 ± 0.22 a

Data are given as mean ± standard deviation (*n* = 3). tr: traces. nd: not detected. Mean values within a row with different letters (a-e) are significantly different (homogenous groups) at *p* ≤ 0.05.

## Data Availability

All related data and methods are presented in this paper. Additional inquiries should be addressed to the corresponding authors.

## Supplementary Materials

The following supporting information can be downloaded at: https://www.mdpi.com/article/10.3390/antiox12040805/s1, Table S1a,b: Spearman correlation coefficients and significance level; Table S2: Quantification of phenolic compounds by UPLC-PDA method (mg/100 g fw).
