# Supplementary material for: Antioxidant Activity and Inhibition of Digestive Enzymes of New Strawberry Tree Fruit/Apple Smoothies
Source: Antioxidants (Basel). 2023 Mar 26;12(4):805. doi: 10.3390/antiox12040805 (PMC10135069; doi:10.3390/antiox12040805)
Supplement: Supplementary file 1 [file antioxidants-12-00805-s001.zip › antioxidants-2283501-supplementary.pdf]

Table S1a. Spearman correlation coefficients and significance level ($p \leq 0.05$ and $p \leq 0.01$).

	TP (Folin-Chiocalteus' assay)	CUPRAC	FRAP	ORAC	$DPPH^{\bullet}$	$ABTS^{\bullet+}$	*α*-amylase	*α*-glucosidase	pancreatic lipase	Total anthocyanins	Total hydroxybenzoic acids	Total hydroxycinnamic acids	Total dihydrochalcones	Total flavan-3-ols	Polymeric proanthocyanidins	Total flavonols	Total polyphenols (UPLC-PDA)
**CUPRAC**	0.9670																
**FRAP**	0.8161	0.6800															
**ORAC**	0.3717	0.4232	0.4734														
**$DPPH^{\bullet}$**	0.6908	0.6052	0.9293	0.6139													
**$ABTS^{\bullet+}$**	0.8181	0.6931	0.9508	0.2653	0.8982												
***α*-amylase**	-0.9569	-0.9112	-0.7984	-0.4827	-0.6316	-0.7141											
***α*-glucosidase**	-0.7823	-0.8338	-0.7135	-0.6766	-0.8345	-0.7226	0.6868										
**pancreatic lipase**	0.9359	0.9890	0.6179	0.4881	0.5432	0.5981	-0.9127	-0.8099									
**Total anthocyanins**	0.5877	0.6952	0.0733	-0.2265	-0.0052	0.2567	-0.4294	-0.3987	0.6708								
**Total hydroxybenzoic acids**	0.3345	0.1649	0.7572	0.1382	0.8091	0.8133	-0.2228	-0.4244	0.0477	-0.2205							
**Total hydroxycinnamic acids**	0.2884	0.2737	0.4188	-0.0286	0.6017	0.6338	-0.0294	-0.5655	0.1531	0.2720	0.7261						
**Total dihydrochalcones**	-0.4607	-0.3020	-0.8818	-0.5573	-0.9059	-0.7797	0.4818	0.5408	-0.2463	0.3783	-0.8557	-0.3899					
**Total flavan-3-ols**	0.3754	0.2096	0.7609	0.0889	0.8028	0.8399	-0.2450	-0.4416	0.0861	-0.1363	0.9958	0.7625	-0.8220				
**Polymeric proanthocyanidins**	0.3038	0.1331	0.7548	0.1927	0.8162	0.7883	-0.2101	-0.4164	0.0231	-0.2914	0.9964	0.6887	-0.8843	0.9846			
**Total flavonols**	0.2596	0.2657	-0.1854	-0.6141	-0.4714	-0.0819	-0.2412	0.2792	0.2649	0.6870	-0.4580	-0.3009	0.5735	-0.3905	-0.5183		

**Total polyphenols (UPLC-PDA)**	0.5741	0.4273	0.7698	-0.0790	0.7380	0.9125	-0.4007	-0.4919	0.2939	0.2189	0.8913	0.7891	-0.6545	0.9278	0.8520	-0.0514	
**Fructose**	0.6428	0.5760	0.3264	-0.4350	0.0384	0.4466	-0.5720	-0.1023	0.5230	0.7319	0.0268	0.0612	0.1033	0.1007	-0.0405	0.8544	0.4391
**Sorbitol**	-0.0995	-0.3335	0.2461	-0.2429	0.0031	0.1317	-0.0442	0.4962	-0.3609	-0.5490	0.3144	-0.3445	-0.3743	0.2837	0.3338	0.0782	0.1884
**Glucose**	0.1274	-0.0366	0.4943	-0.2617	0.5320	0.6511	0.0480	-0.1646	-0.1782	-0.1139	0.9115	0.7832	-0.5745	0.9278	0.8857	-0.2555	0.8838
**Sucrose**	0.1251	0.2848	-0.3806	-0.4614	-0.3699	-0.1482	0.0592	-0.0783	0.2693	0.8727	-0.4277	0.2333	0.7081	-0.3495	-0.4955	0.6203	-0.0452
**Total sugars**	0.6326	0.5386	0.3965	-0.4743	0.1277	0.5429	-0.5310	-0.1214	0.4612	0.6811	0.1915	0.2043	-0.0001	0.2657	0.1219	0.7747	0.5826
**Oxalic acid**	0.4779	0.2853	0.8500	0.1224	0.8202	0.8870	-0.3930	-0.4278	0.1716	-0.1389	0.9727	0.6258	-0.8723	0.9750	0.9639	-0.2950	0.9211
**Citric acid**	0.3172	0.2921	0.2772	0.5996	0.1155	-0.0065	-0.5763	-0.1014	0.3954	-0.2116	-0.2755	-0.7141	-0.2366	-0.3180	-0.2284	0.0289	-0.3677
**Isocitric acid**	-0.0355	-0.0213	0.0769	-0.2699	0.2902	0.3336	0.3103	-0.2822	-0.1371	0.2163	0.5474	0.9329	-0.1107	0.5842	0.5075	-0.2578	0.5995
**Malic acid**	0.2057	0.2814	0.2431	0.9496	0.3490	-0.0095	-0.3802	-0.4572	0.3842	-0.2874	-0.1422	-0.3229	-0.3430	-0.1999	-0.0791	-0.5108	-0.3711
**Quinic acid**	0.6540	0.5645	0.7962	0.1304	0.8412	0.9325	-0.4648	-0.7090	0.4458	0.2988	0.8562	0.8696	-0.6687	0.8916	0.8211	-0.1719	0.9562
**Shikimic acid**	0.1553	0.2345	0.1676	0.9083	0.2512	-0.0968	-0.3507	-0.3693	0.3482	-0.2982	-0.2358	-0.4269	-0.2653	-0.2947	-0.1714	-0.4501	-0.4596

**Total organic acids**	0.4543	0.3796	0.6811	0.1389	0.8097	0.8267	-0.2489	-0.6555	0.2586	0.1473	0.8894	0.9436	-0.6616	0.9130	0.8638	-0.3693	0.9045
**Sugar/organic acids**	0.0475	0.0213	-0.2872	-0.5245	-0.6126	-0.3021	-0.1514	0.5318	0.0610	0.3320	-0.5842	-0.6639	0.5482	-0.5507	-0.6138	0.8977	-0.3164

Table S1b. Spearman correlation coefficients and significance level ($p \leqslant 0.05$ and $p \leqslant 0.01$).

	Fructose	Sorbitol	Glucose	Sucrose	Total sugars	Oxalic acid	Citric acid	Isocitric acid	Malic acid	Quinic acid	Shikimic acid	Total organic
**Sorbitol**	0.1666											
**Glucose**	0.1312	0.3047										
**Sucrose**	0.4658	-0.6711	-0.1801									
**Total sugars**	0.9827	0.2154	0.3122	0.4181								
**Oxalic acid**	0.2111	0.4212	0.8645	-0.4302	0.3586							
**Citric acid**	0.0107	0.3065	-0.5926	-0.4942	-0.1071	-0.1348						
**Isocitric acid**	-0.0518	-0.3854	0.7275	0.3555	0.0891	0.4035	-0.9112					
**Malic acid**	-0.4859	-0.2048	-0.5228	-0.4563	-0.5706	-0.1451	0.7377	-0.5053				
**Quinic acid**	0.3321	-0.0649	0.7883	0.0243	0.4611	0.8590	-0.3474	0.6505	-0.1812			
**Shikimic acid**	-0.4764	-0.1728	-0.6029	-0.4482	-0.5760	-0.2290	0.7818	-0.5887	0.9935	-0.2842		
**Total organic acids**	0.1020	-0.1255	0.8430	-0.0264	0.2518	0.8321	-0.4832	0.7792	-0.1776	0.9644	-0.2876	
**Sugar/organic acids**	0.6526	0.3501	-0.4399	0.2907	0.5464	-0.4079	0.3188	-0.6007	-0.3248	-0.4837	-0.2325	-0.6681

Table S2. Quantification of phenolic compounds by UPLC-PDA method (mg/100 g fw).

Code	Compound	R_t (min)	λ_{max} (nm)	MS $[M-H]^-$ (m/z)*	MS/MS $[M-H]^-$ (m/z)	Smoothie composition (mg/100 g fw) Au/Md	Au/Md+Cs	Au/Md+Mc	Au/Md+As	Au/Md+Dk
Anthocyanins										
A1	Delphinidin-3,5-*O*-diglucoside	3.040	518	627.2785^+	465.1909/303.1100	nd	0.35±0.02a	nd	nd	nd
A2	Delphinidin-3-*O*-galactoside	3.664	522	465.1909^+	303.1100	nd	nd	0.44±0.03a	nd	nd
A3	Delphinidin-3-*O*-glucoside	3.856	515	465.1953^+	303.1100	nd	nd	8.03±0.22a	nd	nd
A4	Cyanidin-3-*O*-galactoside	4.135	515	449.1959^+	287.1116	1.25±0.05c	1.79±0.11b	3.16±0.11a	1.04±0.11d	1.23±0.10cd
A5	Cyanidin-3-*O*-glucoside	4.397	520	449.1959^+	287.1116	nd	nd	2.34±0.15b	2.79±0.14a	nd
A6	Cyanidin-3-*O*-arabinoside	4.629	515	419.1797^+	287.1116	0.22±0.02a	nd	nd	0.04±0.00b	0.06±0.01c
A7	Petunidin-3-*O*-glucoside	4.648	525	479.2150^+	317.1295	nd	0.03±0.00a	0.65±0.06b	nd	nd
A8	Peonidin-3-*O*-glucoside	5.156	525	463.2164^+	301.1330	nd	nd	0.03±0.01a	nd	nd
A9	Malvidin-3-*O*-glucoside	5.391	519	493.2339^+	331.1495	nd	nd	10.47±0.44a	nd	nd
Total						**1.47±0.03d**	**2.17±0.07c**	**25.12±0.18a**	**3.87±0.05b**	**1.29±0.06e**
Hydroxybenzoic acids										
B1	Gallic acid glucoside I	1.156	280	331.1266	271.1605/169.1417	3.91±0.04b	3.34±0.12d	3.75±0.06c	4.71±0.11a	3.25±0.10d
B2	Galloyl glucoside I	1.274	277	331.1334	169.0417	7.84±0.11b	6.69±0.21c	7.51±0.24b	9.44±0.42a	6.52±0.15c
B3	Gallic acid glucoside II	1.366	270	331.1334	271.1990/169.1417	1.68±0.12ab	1.62±0.09ab	1.71±0.02a	1.64±0.03b	1.80±0.10b
B4	3-*O*-Galloylquinic acid (Theogallin)	1.551	273	343.0742	191.1410	73.86±1.22a	66.75±1.03c	69.94±0.42b	73.53±0.34a	66.42±1.23c
B5	Galloyl glucoside III	1.627	273	331.0639	169.0117	nd	nd	nd	nd	2.01±0.22a
B6	Gallic acid 4-*O*-*β*-D-glucopyranoside	2.080	320	331.1334	169.0417	0.10±0.00a	0.41±0.06b	0.10±0.01ac	nd	0.08±0.01c
B7	Castalagin	2.084	280	933.1019	785.1813/481.0917/301.1057	nd	nd	nd	4.26±0.42a	nd
B8	Galloyl shikimic acid	2.314	272	325.0878	169.0417/125.4180	2.74±0.11b	2.33±0.08c	2.73±0.12b	3.37±0.11a	2.55±0.17bc
B9	Casuarin	2.420	374	783.1445	481.0421/301.0667	nd	nd	nd	1.69±0.08a	nd

B10	Digalloylquinic acid I	2.755	273	495.1837	343.1255/191.3072	1.15±0.11a	0.99±0.05a	0.23±0.01b	0.12±0.01c	0.26±0.04b
B11	Ellagitannin II	2.829	270	933.1114	781.0445/633.0131/301.1057	nd	nd	nd	15.28±0.22a	nd
B12	Quinic acid 3,5-di-*O*-gallate	2.950	273	495.1843	343.0666/325.0878/191.3072	nd	nd	0.31±0.05a	nd	nd
B13	Ellagitannin III	3.035	275	783.0645	481.0186/301.1021	nd	nd	0.11±0.02a	nd	nd
B14	Digalloylquinic acid II	3.210	276	495.0435	343.1158/191.3100	nd	nd	nd	2.26±0.11a	nd
B15	Ellagitannin IV	3.331	280	783.0759	481.0917/301.0667	nd	nd	nd	0.71±0.12a	nd
B16	Nilocitin	3.653	270	481.0938	301.0667/257.1438	nd	nd	nd	0.41±0.05a	nd
B17	Digalloyl shikimic acid I	4.129	278	477.0493	325.0808/169.0417	2.61±0.34ab	2.92±0.11a	0.80±0.09d	1.72±0.10c	2.46±0.06b
B18	Casuarinin	4.391	278	935.0146	765.1799/545.1230	nd	nd	nd	0.28±0.04a	nd
B19	Digalloyl shikimic acid II	4.618	275	477.0493	325.0808/169.0417	3.02±0.22a	3.39±0.43a	nd	nd	3.12±0.21a
B20	Gallotannin derivative	4.869	278	1109.1115	972.1873/635.1085/301.1021	nd	0.43±0.03a	nd	nd	nd
B21	Salicylic acid	5.037	320	136.1212		nd	nd	nd	nd	0.08±0.00a
B22	Ellagic acid arabinoside	5.703	359	433.0224	301.0631	2.91±0.42b	nd	4.81±0.44a	0.92±0.13c	3.65±0.33b
B23	Ellagic acid xyloside	5.885	361	433.0735	301.0631	nd	nd	nd	0.88±0.11a	0.74±0.11a
B24	Ellagic acid	6.028	366	300.0631		nd	nd	nd	4.30±0.31a	0.81±0.12b
Total						**99.84±0.31b**	**88.87±0.24e**	**91.99±0.21d**	**125.51±0.34a**	**93.74±0.13c**
Hydroxycinnamic acids										
C1	Neochlorogenic acid	3.489	317	353.1287	191.3100/136.0212	0.35±0.05b	0.22±0.00c	0.48±0.04a	0.46±0.02a	0.32±0.01b
C2	Chlorogenic acid	3.723	323	353.0838	191.0534	4.57±0.32a	4.28±0.30a	4.47±0.11a	4.43±0.22a	4.27±0.31a
C3	Caffeic acid	4.036	320	311.0807	179.1098	0.24±0.04bc	0.25±0.02b	0.29±0.02b	0.41±0.05a	0.24±0.02c
C4	*p*-Coumaric acid	4.463	323	163.0349		0.14±0.02b	0.18±0.01a	0.08±0.00d	0.10±0.01c	0.18±0.03ab
C5	*p*-Coumaroyloquinic acid	4.904	310	337.0912	163.1014	0.77±0.13a	0.72±0.11a	0.70±0.10a	0.76±0.09a	0.76±0.04a
Total						**6.07±0.13a**	**5.65±0.11b**	**6.02±0.06ab**	**6.16±0.11a**	**5.77±0.22b**
Dihydrochalcones										
D1	Phloretin-2'-*O*-xyloglucoside	7.433	280	567.1703	273.0757	1.79±0.03a	1.87±0.11a	1.84±0.11a	1.42±0.03c	1.71±0.01b

D2	Phloretin-2'-*O*-glucoside (Phloridzin)	8.186	280	435.1332	273.0733	2.20±0.11a	2.17±0.22a	2.22±0.15a	2.05±0.11a	2.03±0.11a
Total						**3.99±0.10a**	**4.04±0.12a**	**4.06±0.11a**	**3.47±0.08c**	**3.74±0.10b**
Flavan-3-ols										
E1	Procyanidin B1	3.256	280	577.1293	289.0708	6.70±0.42c	8.60±0.32b	19.36±0.15a	2.20±0.04e	3.08±0.02d
E2	Procyanidin B3	3.562	277	577.1055	289.2014/245.2391	0.06±0.00d	0.13±0.00c	0.38±0.01b	0.90±0.11a	0.06±0.00d
E3	(+)-Catechin	3.669	280	289.0673	245.0780	10.96±0.24c	10.79±0.11c	11.54±0.08b	13.03±0.54a	10.14±0.21d
E4	(-)-Epiatechin	4.143	280	289.0673	245.0780	6.64±0.22ab	6.83±0.11a	1.05±0.06d	6.28±0.16bc	6.10±0.11c
E5	Procyanidin B2	4.631	280	577.1293	289.0708	0.91±0.04d	1.18±0.01b	3.47±0.14a	1.12±0.03c	1.14±0.04bc
E6	Procyanidin C1	4.956	280	866.1908	577.1188/289.0708	0.44±0.01d	0.49±0.00c	0.71±0.04a	0.44±0.00d	0.56±0.03b
Total						**434.33±3.12b**	**412.44±2.55d**	**423.64±5.44c**	**487.37±4.13a**	**419.63±4.45c**
PP#	Polymeric proanthocyanidins					408.62±9.31b	384.42±5.42c	387.13±6.81c	463.40±3.12a	398.55±4.12b
DP	Degree of polymerisation					4.61	4.58	4.67	4.95	4.81
Flavonols										
F1	Kaempherol-3-*O*-sophoroside-7-*O*-glucoside	3.546	346	771.0181	609.0240/285.1257	nd	0.34±0.02a	nd	nd	nd
F2	Kaempferol-3,7-*O*-diglucoside	4.636	345	609.0341	447.0543/285.1292	nd	0.12±0.00a	nd	nd	nd
F3	Isorhamnetin-3,7-*O*-digalactoside	4.747	350	639.0883	447.0327/315.0605	nd	0.75±0.12a	nd	nd	nd
F4	Quercetin derivative I	4.766	359	633.0900	463.0633/301.0667	0.36±0.03b	nd	0.41±0.02a	0.46±0.06a	0.30±0.02c
F5	Myricetin galactoside-gallate	4.995	360	631.1107	479.1073/317.1114	nd	nd	1.35±0.12a	nd	nd
F6	Quercetin-3,7-*O*-diglucoside	5.356	352	625.1044	463.0333/301.0924	nd	1.80±0.11a	nd	0.10±0.00b	nd
F7	Myricetin-3-*O*-galactoside	5.460	356	479.0610	317.1114	0.11±0.01e	0.25±0.02b	9.32±0.23a	0.16±0.02c	0.13±0.00d
F8	Myricetin-3-*O*-glucoside	5.534	356	479.0162	317.1114	0.10±0.00c	0.86±0.03a	0.48±0.05b	nd	0.04±0.00d
F9	Isorhametin-3,7-*O*-diglucoside	5.709	343	639.1035	477.1314/315.0948	nd	1.66±0.12a	nd	nd	nd
F10	Quercetin galloylhexose	5.816	360	615.1291	463.0950/301.1092	0.26±0.01c	0.30±0.03b	0.51±0.15a	nd	0.31±0.02b
F11	Quercetin-3-*O*-rutinoside	5.957	359	609.1419	301.0319	nd	nd	nd	nd	0.86±0.04a
F12	Kaempferol-3-*O*-sophoroside	5.978	358	609.0240	285.1226	nd	9.57±0.16a	nd	nd	nd

F13	Myricetin-3-*O*-arabinoside	6.027	364	449.0362	317.0678	nd	nd	1.06±0.02a	nd	nd
F14	Quercetin derivative II	6.053	366	633.1003	463.0861/301.1021	0.21±0.00a	nd	nd	nd	nd
F15	Myricetin-3-*O*-xyloside	6.161	350	449.1883	317.1114	0.30±0.02a	0.32±0.02a	0.31±0.01a	0.24±0.02b	0.24±0.01b
F16	Myricerin-3-*O*-rhamnoside	6.239	347	463.0861	317.1114	0.23±0.01b	0.12±0.01d	5.78±0.15a	0.18±0.00c	0.12±0.01d
F17	Quercetin-3-*O*-galactoside	6.345	348	463.0861	301.0994	0.92±0.06c	0.88±0.04c	1.34±0.01b	1.65±0.11a	0.93±0.03c
F18	Quercetin-3-*O*-glucoside	6.494	350	463.1774	301.1447	0.26±0.02c	0.49±0.02a	0.40±0.02b	0.45±0.04ab	0.23±0.00d
F19	Kaempferol-3-*O*-galactoside	6.640	347	447.0629	285.1326	nd	nd	nd	0.55±0.04a	nd
F20	Isorhamnetin-3-*O*-rutinoside	6.697	352	623.1223	315.0871	nd	1.01±0.06	nd	nd	nd
F21	Quercetin-3-*O*-arabinoside	6.765	350	433.0160	301.1421	0.11±0.00c	0.14±0.01b	0.17±0.02b	0.27±0.04d	0.22±0.02c
F22	Quercetin-pentoside	6.957	360	433.0565	301.0959	nd	nd	nd	0.50±0.05a	0.11±0.00
F23	Quercetin-3-*O*-xyloside	7.063	350	433.0160	301.1421	1.31±0.12a	1.37±0.06a	1.35±0.07a	1.43±0.05a	1.29±0.15a
F24	Quercetin-3-*O*-rhamnoside	7.325	348	447.0148	301.1447	1.73±0.11c	2.54±0.20a	2.19±0.13b	1.52±0.21d	1.77±0.13a
F25	Isorhanetin-3-*O*-glucoside	7.539	352	477.1314	315.0871	nd	0.07±0.00a	nd	nd	nd
F26	Kaempferol-hexoside I	7.568	350	447.1543	285.1326	nd	nd	nd	0.15±0.01a	nd
F27	Myricetin	7.683	367	317.1114		nd	nd	0.36±0.03a	nd	nd
F28	Quercetin	8.774	360	301.1065		nd	nd	nd	0.12±0.01a	nd
F29	Kaempferol	9.435	360	285.1706		nd	nd	nd	1.80±0.12a	nd
Total						**5.90±0.12e**	**22.59±0.14b**	**25.03±0.22a**	**9.58±0.13c**	**6.55±0.09d**
**Total phenolic compounds** (including polymeric proanthocyanidins)						**551.60±4.51c**	**535.75±3.11d**	**575.86±1.66b**	**635.96±5.21a**	**530.72±6.11d**

Data are given as mean ± standard deviation ($n = 3$). nd: not detected. Mean values within a line with different letters (a-e) are significantly different (homogenous groups) at $p \leq 0.05$. * $[M+H]^+$ (*m/z*) for anthocyanins were obtained in the positive ion mode. # Quantitative data of polymeric proanthocyanidins were obtained using the phloroglucinol method.
